# Thyroid Dysfunction in Patients With End-Stage Renal Disease: A Single-Centered Experience From Pakistan

**DOI:** 10.7759/cureus.76715

**Published:** 2025-01-01

**Authors:** Sidra German, Sajid Bhatti, Tajammul Waqar, Sajid Lashari, Maria Mehmood, Anum Rizwan, Huraira Ali, Raja Taha Yaseen

**Affiliations:** 1 Nephrology, Sindh Institute of Urology and Transplantation, Karachi, PAK; 2 Medicine, Jinnah Sindh Medical University, Karachi, PAK; 3 Gastroenterology, Sindh Institute of Urology and Transplantation, Karachi, PAK

**Keywords:** end-stage renal disease (esrd), hypothyroidism, pakistan, prevalence, thyroid

## Abstract

Introduction

The global prevalence of chronic kidney disease (CKD) is increasing due to diabetes and hypertension, with a substantial number of patients progressing to end-stage renal disease (ESRD) requiring maintenance dialysis. Thyroid dysfunction, particularly hypothyroidism, is common in CKD and ESRD patients but is often underdiagnosed due to symptom overlap with uremia. This study aimed to determine the prevalence of thyroid dysfunction in hemodialysis patients at the Sindh Institute of Urology and Transplantation (SIUT), Karachi.

Methods

A prospective cross-sectional study was conducted, enrolling 131 patients on maintenance hemodialysis for at least three months. Participants' demographic and clinical data were collected, and blood samples were taken for thyroid function testing using an Access hypersensitive thyroid-stimulating hormone (hTSH) immunoassay system. Statistical analyses were performed using SPSS version 22.0 (IBM Corp., Armonk, NY), with a significance threshold of p < 0.05.

Results

The total population included in the study was 131. Out of them, 74 (56.5%) were males. The study population had a mean age of 56.4 years. Hypertension and diabetes were the most common comorbidities noticed in 73 (55.7%) and 14 (10.7%) patients, respectively. Hypothyroidism was observed in 27 (20.6%) patients, with 18 (66.7%) of them having overt hypothyroidism. Hyperthyroidism was observed in four (3.1%) patients, all subclinical. Significant risk factors for hypothyroidism included female gender, longer duration on hemodialysis, decreased total leukocyte and platelet counts, and increased serum urea and creatinine levels.

Conclusion

The study underscores the significant burden of thyroid dysfunction in ESRD patients on hemodialysis, emphasizing the need for regular screening and management to mitigate associated cardiovascular risks and improve patient outcomes. Future studies should explore the long-term benefits of routine thyroid screening and the impact of different dialysis modalities on thyroid function.

## Introduction

The prevalence of chronic kidney disease (CKD) has been on a rising trend due to diabetes and hypertension [1]. It has been perceived as a public health problem [2]. Approximately 2.5 million people are receiving renal replacement therapy, and it is estimated to double by 2030 to 5.4 million. However, due to the lack of availability of renal replacement facilities in many countries, approximately 2.3 - 7.1 million adults have died prematurely due to the lack of renal replacement therapy [3].

Thyroid-stimulating hormone (TSH) is secreted by the anterior pituitary gland, which stimulates the thyroid gland, located in the front of the neck to secrete thyroid hormones thyroxine (T4) and triiodothyronine (T3). T3 is a metabolically active form and reverse (rT3) is an inactive form also produced from the peripheral organs including the kidney. However, T4 is only produced by the thyroid gland. Only a small fraction of the thyroid hormones circulate as free hormones, acting on the target tissues, whereas approximately 99% of these hormones are tightly bound to the carrier proteins, namely thyroid binding globulin (TBG), transthyretin, and albumin [4].

Endocrine dysfunction such as thyroid problems is quite common among CKD and end-stage renal disease (ESRD) patients or those who are on maintenance dialysis. It is one of the most underappreciated endocrine disorders in patients with CKD or ESRD patients. Hypothyroidism is a state in which TSH is increased and it can be either subclinical or overt type depending upon the free T4 (FT4) levels in the serum. Overt hypothyroidism is the most prevalent thyroid disorder in ESRD patients and if remains untreated, it further exacerbates the cardiovascular disease, which is highly prevalent and the leading cause of mortality among ESRD patients [5,6].

The alteration in the iodine metabolism in the body due to the compromised renal function in CKD and ESRD patients results in hypothyroidism and hyperthyroidism via the Wolf Chaikoff effect and Jod Basedow phenomenon respectively [7]. It is proposed that thyroid dysfunction in CKD patients is not due to the primary gland dysfunction, rather it is due to the non-thyroidal illness. Various factors, like malnutrition, uremia, and chronic metabolic acidosis, affect not only iodothyronine de-iodination but also as protein binding of T3, reducing the peripheral conversion of T4 to T3 and its protein binding [8]. Due to the loss of thyroid-binding proteins and minerals like selenium in dialysis-dependent populations and alterations in the central hypothalamic-pituitary axis, it is found that TSH is insignificantly raised with low T4 [7]. According to the Asian and US hemodialysis and peritoneal dialysis cohorts, the prevalence of hypothyroidism varies from 13% to 25%. Despite this data, it remains under-recognized or undiagnosed due to the overlap of symptoms with uremia, i.e., cold intolerance, decreased cognition, and fatigue [9]. A study done in Hyderabad, Pakistan, showed a prevalence of 37.9% of hypothyroidism among the hemodialysis population [10].

The aim of our study was to determine the prevalence of thyroid dysfunction in hemodialysis patients. The purpose of this study is to determine the burden of thyroid disorders in dialysis-dependent patients, so that if this burden is large enough, this needs to establish a proper screening program, in this population for early detection and timely management.

## Materials and methods

Study design and study place

This cross-sectional study was carried out at the Department of Nephrology, Sindh Institute of Urology and Transplantation (SIUT) synopsis from January 1, 2023 to June 30, 2023. Considering the total population of hemodialysis patients is (N = 2,000), taking the expected prevalence of hypothyroidism as 16% with a margin of error of 5% and 95% confidence interval. A sample size of 120 patients was calculated for this study. The patients were recruited through the method of non-probability consecutive sampling.

Inclusion and exclusion criteria

The study included patients who were older than 18 years of either gender, with a glomerular filtration rate (GFR) less than 15 mL/min, on maintenance dialysis for at least three months and had permanent angio-access or a tunneled dialysis catheter. Excluded from the study were patients with acute illness, known thyroid disorders, those taking thyroid hormones, those with a history of thyroidectomy or family history of thyroid disorders, and patients taking medications known to affect thyroid function, such as amiodarone, glucocorticoids (over 50 mg/day), lithium, and phenytoin beta blockers. Additionally, patients who had recently (within two weeks) undergone contrast imaging were also excluded.

Data collection

After the informed consent, all the patients fulfilling the inclusion criteria were enrolled in the study. The demographic data including the age, gender, weight, clinical history of comorbidities like diabetes and hypertension, cause of renal failure, frequency, and duration (number of years) of hemodialysis were recorded.

Blood samples were obtained from all the patients in the fasting state who were coming for maintenance hemodialysis through arterio-venous fistula cannulation (or from a venous catheter in those who had it), before heparin administration in the morning. Thyroid hormone testing was done at the laboratory of SIUT using an Access hypersensitive hTSH immunoassay system. It is a solid-phase immunoenzymatic assay with virtually no cross-reactivity to other peptide hormones. The sensitivity of this test enables better discrimination between hyperthyroid and euthyroid patients (Table 1).

**Table 1:** Operational definitions of thyroid dysfunction and its types TSH: Thyroid-stimulating hormone; FT4: Free thyroxine

Operational Definitions:
Thyroid dysfunction [11]: When a human body produces too much or too little thyroid hormone, it is called thyroid dysfunction. Overt and subclinical hypothyroidism definition was adopted based on the American Thyroid Association guidelines 2012 and the European Thyroid Association guidelines ATA/AACE 2013.
Hypothyroidism [11]: It is defined as TSH level of > 5.6 mIU/mL+ normal or low FT4 and categorized based on FT4 as overt hypothyroidism, if low FT4 (<12 pmol/L) and high TSH, and subclinical hypothyroidism, if high TSH and normal FT4.
Overt hyperthyroidism [11]: It is defined as TSH lower than 0.22 μIU/mL and FT4 higher than 22 μIU/mL, and subclinical hyperthyroidism was low TSH and normal FT4.

TSH testing was done for all the patients, and FT4 was done for those who have either high or low TSH levels for further categorization into subclinical or overt type. Thyroid autoantibodies (anti-thyroid peroxidase antibodies) were also checked in those patients who had evidence of hypothyroidism.

Data analysis procedure

The data were entered and analyzed using the Statistical Package for Social Sciences (SPSS) version 22.0 (IBM Corp., Armonk, NY). Mean ± Standard Deviation (S.D.) was used for the expression of continuous variables while categorical variables were expressed as frequencies and percentages. Continuous variables were analyzed using the student t-test while the chi-square test was utilized for the stratification of categorical variables. The outcome was noted in terms of the presence or absence of hypothyroidism and was further categorized into overt and subclinical types according to the American Thyroid Association guidelines 2012 and the European Thyroid Association guidelines ATA/AACE 2013. Hyperthyroidism was excluded from the stratification analysis due to its lower frequency in the ESRD population. A p-value of <0.05 was considered statistically significant.

## Results

A total of 131 patients were enrolled in the study. Baseline characteristics are shown in Table 2. Among them, 74 (56.5%) were males. The mean age was 56.4±3.6 years. The most common comorbid condition in the studied population was hypertension observed in 73 (55.7%) patients followed by diabetes in 14 (10.7%) patients. Among the studied population, 16 (12.2%) patients were smokers. A most common cause of CKD in our population was hypertension observed in 73 (55.7%) followed by diabetes in 14 (10.7%), adult polycystic kidney disease in eight (6.1%), chronic glomerulonephritis in seven (5.3%), small-sized kidneys in seven (5.3%), bladder outlet obstruction in six (4.6%), stone disease in five (3.8%), and neurogenic bladder in one (0.8%). Out of 131 patients, 43 (32.8%) patients were on maintenance hemodialysis for more than four years. Most of the patients (109 (83.2%)) had two sessions of hemodialysis per week.

**Table 2:** Baseline characteristics of the population included in the study (N-131) The data have been represented as N, categorical variables as %, and continuous variables as Mean ± SD. CKD: Chronic kidney disease; APKD: Adult polycystic kidney disease; GN: Glomerulonephritis; TSH: Thyroid-stimulating hormone; FT4: Free T4 levels

Study population		N (%)
Gender	Male	74 (56.5)
	Female	57 (43.5)
Comorbidities	Hypertension	73 (55.7)
	Diabetes	14 (10.7)
History of Smoking	Yes	16 (12.2)
	No	115 (87.8)
Cause of CKD	Hypertension	73 (55.7)
	Diabetes	14 (10.7)
	APKD	8 (6.1)
	Chronic GN	17 (13)
	Small Sized Kidneys	7 (5.3)
	Bladder Outlet obstruction	6 (4.6)
	Stone disease	5 (3.8)
	Neurogenic Bladder	1 (0.8)
Duration of Hemodialysis	< 4 years	78 (59.5)
	≥ 4 years	53 (40.5)
Number of hemodialysis sessions per week	<2 sessions	109 (83.2)
	≥2 sessions	23 (16.8)
Thyroid status	Hyperthyroid	4 (3.1)
	hypothyroid	27 20.6)
	Euthyroid	100 (76.3)
Type of hypothyroidism	Overt	18 (66.7)
	Subclinical	9 (33.3)
Age (years± S.D)		56.4 ± 3.6
Hemoglobin (g/dL)		9.28 ± 1.8
Total Leucocyte Count (x10^9^/L)		6.2 ± 3.1
Platelet Count (x10^9^/L)		176 ± 71
Serum Urea (mg/dl)		127.8 ± 40.9
Serum Creatinine (mg/dl)		9.3 ± 2.8
Serum Albumin (g/dl)		3.2 ± 1.2
Alkaline Phosphatase (IU/L)		307 ± 44
Serum Calcium (mg/dl)		8.2 ± 1.0
TSH (mIU/mL)		3.6 ± 3.0
FT4 levels(pg/ml)		10.7 ± 1.8

Mean hemoglobin (Hb) was 9.28±1.8 g/dL, total leucocyte count (TLC) of 6.2±3.1 x10^9^/L, platelet count of 176±71 x10^9^/L, serum creatinine of 9.3±2.8 mg/dL, urea of 127.8±40.9 mg/dL, serum albumin of 3.2±1.2 g/dL, calcium of 8.2±1.0 mg/dL, alkaline phosphatase of 307±44 IU/L, mean TSH of 3.6±3.0 mIU/mL, and FT4 levels of 10.7± 1.8 pg/mL.

In our ESRD population, 27 (20.6%) patients had hypothyroid status, and only four (3.1%) patients had hyperthyroid status while 100 (76.3%) patients had euthyroid status. Of 27 patients with hypothyroidism, 18 (66.7%) patients had over hypothyroidism while nine (33.3%) patients had subclinical hypothyroidism. All four patients with hyperthyroidism had subclinical hyperthyroidism (Table 2).

On comparative analysis, female gender (p≤0.001), ESRD patients on hemodialysis for more than two years (p=0.01), less than three sessions of hemodialysis per week (p=0.05), decreased TLC (p=0.001), platelet count (p=0.035), increased serum urea (p=0.01), and creatinine levels (p=0.042) were the factors that significantly increased the risk of developing hypothyroidism in ESRD population. However, in our study, serum albumin (p=0.478) was not associated with the development of hypothyroidism (Table 3).

**Table 3:** Comparison of baseline variables in terms of hypothyroidism (N-131) The data have been represented as n, categorical variables as % and continuous variables as Mean ± SD and the p-value of ≤0.005 was considered statistically significant.

Variable		Hypothyroidism		P-value
		Yes (n-27); n (%)	No (n-104); n (%)	
Gender	Males	6 (22)	68 (65.3)	≤0.001
	Females	21 (78)	36 (34.7)	
Hemodialysis duration	≥ 2 years	3 (11.1)	75 (72.1)	0.01
	<2 years	24 (88.9)	29 (27.9)	
Frequency of hemodialysis	2 sessions/week	21 (78)	88 (84.6)	0.05
	3 sessions/week	6 (22)	16 (15.4)	
Age (years)		44.8 ± 16.1	45.5 ± 14.4	0.797
Hemoglobin (g/dL)		9.8 ± 1.7	9.2 ± 1.8	0.097
Total leucocyte count (x10^9^/L)		4.4 ± 1.4	6.7 ± 3.3	0.001
Platelet count (x10^9^/L)		151 ± 64	183 ± 71	0.035
Serum urea (mg/dL)		132 ± 42	109 ± 25	0.01
Serum creatinine (mg/dL)		9.5 ± 2.9	8.4 ± 1.7	0.042
Alkaline phosphatase (IU/L)		345 ± 274	296 ± 312	0.314
Serum calcium (mg/dL)		8.2 ± 0.93	8.2 ± 1.1	0.81
Serum albumin (g/dL)		2.9 ± 0.7	3.1 ± 0.9	0.37

## Discussion

The prevalence of CKD is rising globally, driven primarily by diabetes and hypertension, and it has emerged as a significant public health issue [12]. Approximately 2.5 million people currently receive renal replacement therapy, a number projected to more than double by 2030 [13]. Thyroid dysfunction, particularly hypothyroidism, is common among patients with CKD and ESRD, especially those undergoing maintenance dialysis [14]. Despite its prevalence, thyroid dysfunction in this population is often underappreciated and underdiagnosed due to overlapping symptoms with uremia [15].

 The prevalence of hypothyroidism observed in our study is comparable with earlier research. Studies conducted in various cohorts of hemodialysis patients have reported a prevalence of hypothyroidism ranging from 13% to 25% [16,17]. For instance, a study from the United States and Asia noted a similar prevalence, emphasizing the commonality of this condition in ESRD patients [18,19]. However, our findings show a higher prevalence than that reported by Chonchol et al., who found a hypothyroidism prevalence of 16% in hemodialysis patients [14]. Additionally, a study conducted in Hyderabad, Pakistan, reported an even higher prevalence of 37.9% of hypothyroidism among the hemodialysis population, suggesting regional variations might influence the prevalence rates due to different genetic, environmental, and healthcare factors [20].

Our study identified several significant risk factors associated with hypothyroidism in ESRD patients, including female gender, longer duration of hemodialysis (more than two years), decreased number of hemodialysis sessions per week, decreased TLC, lower platelet count, and higher serum urea and creatinine levels. These findings are consistent with previous studies that have highlighted similar risk factors. For example, Carrero et al. found that prolonged duration of dialysis and higher urea levels were significant predictors of thyroid dysfunction [21]. Moreover, the association of hypothyroidism with the female gender has been well-documented in the literature, reflecting the higher incidence of thyroid disorders in females across various populations [22].

The pathophysiology of thyroid dysfunction in CKD and ESRD patients is multifactorial [15]. It is primarily attributed to non-thyroidal illness rather than primary thyroid gland dysfunction [16]. Factors such as malnutrition, uremia, and chronic metabolic acidosis play crucial roles in impairing the peripheral conversion of thyroxine (T4) to its active form, triiodothyronine (T3), and affecting the protein binding of T3 [23]. Dialysis further complicates these issues by causing the loss of thyroid-binding proteins and essential minerals like selenium, which are vital for thyroid function [15]. Additionally, the compromised renal function alters iodine metabolism, contributing to thyroid dysfunction through mechanisms such as the Wolff-Chaikoff effect and the Jod-Basedow phenomenon [24].

Diagnosing thyroid dysfunction in ESRD patients is challenging due to the overlap of symptoms with uremia, such as fatigue, cold intolerance, and decreased cognition [25]. This overlap often leads to under-recognition and under-diagnosis of thyroid disorders in this population [26]. Our study highlights the importance of routine screening for thyroid function in ESRD patients. Early detection and timely management of thyroid dysfunction can mitigate the exacerbation of cardiovascular disease, which is a leading cause of mortality in ESRD patients [27]. Therefore, implementing comprehensive screening programs for thyroid dysfunction in dialysis-dependent patients is crucial for improving clinical outcomes and enhancing the quality of life.

There are certain limitations that can be attributed to our study. Single-centered study design may limit the applicability of the findings of the study to broader populations. Second, non-probability sampling may introduce selection bias, potentially affecting the generalizability of the results. Third, the exclusion of hyperthyroidism from detailed analysis due to its low prevalence limits the understanding of its full impact on the ESRD population. Fourth, the study could have benefitted from a broader range of variables, including more detailed nutritional and medication histories, to better understand their impact on thyroid function.

## Conclusions

This study demonstrates a significant burden of thyroid dysfunction, particularly hypothyroidism, among ESRD patients undergoing hemodialysis. The findings emphasize the need for regular thyroid function screening and timely management in this population to address this underappreciated endocrine disorder. By integrating routine thyroid function tests into the standard care for ESRD patients, healthcare providers can facilitate early detection and treatment of thyroid disorders, thereby potentially reducing cardiovascular risks and improving patient outcomes. Future longitudinal studies are warranted to explore the long-term benefits of routine thyroid screening and the impact of different dialysis modalities on thyroid function in ESRD patients.
